# Metabolism-dependent drug response assessment using a liver-cancer microphysiological system on the kinetic-pump integrated microfluidic plate (KIM-Plate)

**DOI:** 10.1016/j.namjnl.2026.100127

**Published:** 2026-09-05

**Authors:** Kenta Shinha, Hiroko Nakamura, Hiroshi Arakawa, Masaki Nishikawa, Yukio Kato, Yasuyuki Sakai, Hiroshi Kimura

**Affiliations:** a Micro/Nano Technology Center (MNTC), Tokai University, 4-1-1 Kitakaname, Hiratsuka, Kanagawa, Japan 259-1292.; b Department of Regulatory Science, Graduate School of Pharmaceutical Sciences, Nagoya City University, 3-1 Tanabe-dori, Mizuho-ku, Nagoya, Aichi, Japan 467-8603; c Department of Chemical System Engineering, Graduate School of Engineering, The University of Tokyo, 7-3-1, Hongo, Bunkyo-ku, Tokyo, Japan 113-8654; d Faculty of Pharmacy, Institute of Medical, Pharmaceutical and Health Sciences, Kanazawa University, Kakuma-machi, Kanazawa, Ishikawa, Japan 920-1192

**Keywords:** Microphysiological system (MPS), Organs-on-a-chip, Kinetic-pump, Coculture, Drug discovery

## Abstract

Reducing reliance on animal studies while improving human relevance requires new approach methodologies (NAMs) that reproduce dynamic biological interactions and metabolism-dependent drug exposure. Microphysiological systems (MPSs) are promising NAMs that can recapitulate selected human organ functions in vitro. However, the advantages of multi-organ MPSs over conventional culture methods have not been sufficiently validated because direct comparisons between these systems are difficult. Here, we constructed a liver–cancer MPS using a Kinetic-pump integrated microfluidic plate (KIM-Plate) containing two open, standard-well-compatible culture chambers connected by recirculating microchannels. PXB human hepatocytes and MCF7 breast cancer cells were evaluated using well-plate monoculture, conditioned-medium transfer, KIM-Plate monoculture, and dynamic KIM-Plate coculture. Dynamic coculture significantly increased MCF7 proliferation relative to KIM-Plate monoculture, whereas conditioned-medium transfer did not significantly increase proliferation relative to well-plate monoculture. PXB cells produced the active CPT-11 metabolite SN-38 and SN-38 glucuronide under both static and perfusion conditions, demonstrating sequential CPT-11-metabolizing activity in the MPS. MCF7 cells showed greater apparent sensitivity to SN-38 under perfusion than under static conditions. During CPT-11 exposure, the dynamic liver–cancer coculture was estimated to generate a gradually increasing SN-38 exposure profile and a lower SN-38 area under the concentration–time curve than conditioned-medium exposure. Nevertheless, observed MCF7 viability was higher than predicted from cumulative SN-38 exposure alone, suggesting that the temporal exposure profile may also have contributed to the drug response. The KIM-Plate therefore provides a tractable human cell-based NAM for investigating how continuous intercellular interactions, perfusion, and time-dependent metabolism collectively influence drug responses, thereby providing mechanistically informative evidence for metabolism-dependent drug assessment.

## 1 Introduction

In drug discovery, accurately assessing the efficacy and toxicity of candidate compounds during nonclinical development remains challenging, and many drug candidates fail in clinical trials because of insufficient efficacy or unacceptable toxicity (Quan et al., 2018; Scott et al., 2013). The increasing complexity of therapeutic modalities further highlights the need for more predictive nonclinical models. Accordingly, new approach methodologies (NAMs), including in silico approaches and microphysiological systems (MPSs), are gaining attention as complements or alternatives to conventional two-dimensional cell culture and animal testing (Avila et al., 2020; Ram et al., 2022). Regulatory interest in NAMs is also increasing, reflecting the need for more human-relevant evidence in drug development. In silico approaches use computational models to predict drug properties and biological responses, whereas MPSs reproduce selected aspects of tissue and organ function in vitro by controlling cellular organization, fluid flow, and other microenvironmental factors (Kimura et al., 2025; Kimura et al., 2018; Wang et al., 2023). MPSs can therefore be used to investigate absorption, distribution, metabolism, and excretion (ADME), as well as pharmacodynamic responses, under controlled experimental conditions. Because drug efficacy and toxicity depend not only on the administered dose but also on the magnitude and temporal profile of drug exposure, reproducing pharmacokinetically relevant exposure conditions is important for in vitro drug evaluation.

To address this need, numerous multi-organ MPSs (MO-MPSs) have been developed to investigate organ–organ interactions and coupled pharmacokinetic–pharmacodynamic responses (Lee and Sung, 2017; Van Berlo et al., 2021; Ronaldson-Bouchard and Vunjak-Novakovic, 2018; Arakawa et al., 2020). In particular, MPSs combining a liver model with a disease model have been used to evaluate how hepatic metabolism affects drug concentrations, efficacy, and toxicity (Sung et al., 2010; Lee et al., 2017). MO-MPSs can also be coupled with mathematical pharmacokinetic–pharmacodynamic models by adjusting system parameters such as flow-rate ratios between organ models, medium residence times, cell numbers, and medium volumes (Abaci and Shuler, 2015). Our group previously developed an intestine–liver–cancer MPS for evaluating the anticancer effects of orally administered drugs while accounting for intestinal absorption and hepatic metabolism (Kimura et al., 2015). We also demonstrated that integration of a liver–cancer MPS with a pharmacokinetic–pharmacodynamic model enabled the estimation of model parameters relevant to drug–drug interaction studies (Shinha et al., 2020).

However, most previous MO-MPS studies investigating metabolism-dependent drug efficacy have focused on determining whether the inclusion of ADME organ models, such as the liver or intestine, alters drug responses. It remains unclear what additional information simultaneous and continuous coculture provides compared with conventional conditioned medium (CM) transfer methods. Direct comparison is challenging because MO-MPSs and conventional well-plate cultures generally differ not only in organ coupling but also in culture geometry, fluid dynamics, medium volume, and analytical accessibility. Consequently, a platform that allows dynamic multi-organ coculture while retaining compatibility with conventional well-plate procedures is needed to investigate how continuous organ–organ interactions and time-dependent drug exposure influence cellular responses.

In this study, we used our previously developed Kinetic-pump integrated Microfluidic Plate (KIM-Plate) to directly compare dynamic liver–cancer coculture with conventional culture methods. The KIM-Plate contains six independent MO-MPS units, each comprising two open, 24-well-sized culture chambers connected by microchannels. This design enables recirculating coculture while allowing the two cell populations to be handled and analyzed separately, thereby facilitating comparison with conventional well-plate methods (Shinha et al., 2021; Kurniawan et al., 2024). Using PXB human hepatocytes, MCF7 breast cancer cells, the prodrug CPT-11, and its active metabolite SN-38, we compared static monoculture, CM method, and simultaneous perfusion coculture. Specifically, we evaluated liver–cancer interactions, hepatic metabolism of CPT-11, and the effects of static versus time-dependent drug exposure on cancer cell responses. Through these comparisons, we examined whether dynamic coculture using the KIM-Plate captures biological and pharmacological responses that are not adequately reproduced by conventional CM methods.

## 2 Results and discussion

### 2.1 Coculture with hepatocytes promotes the proliferation of breast cancer cells

**Fig. 1:**
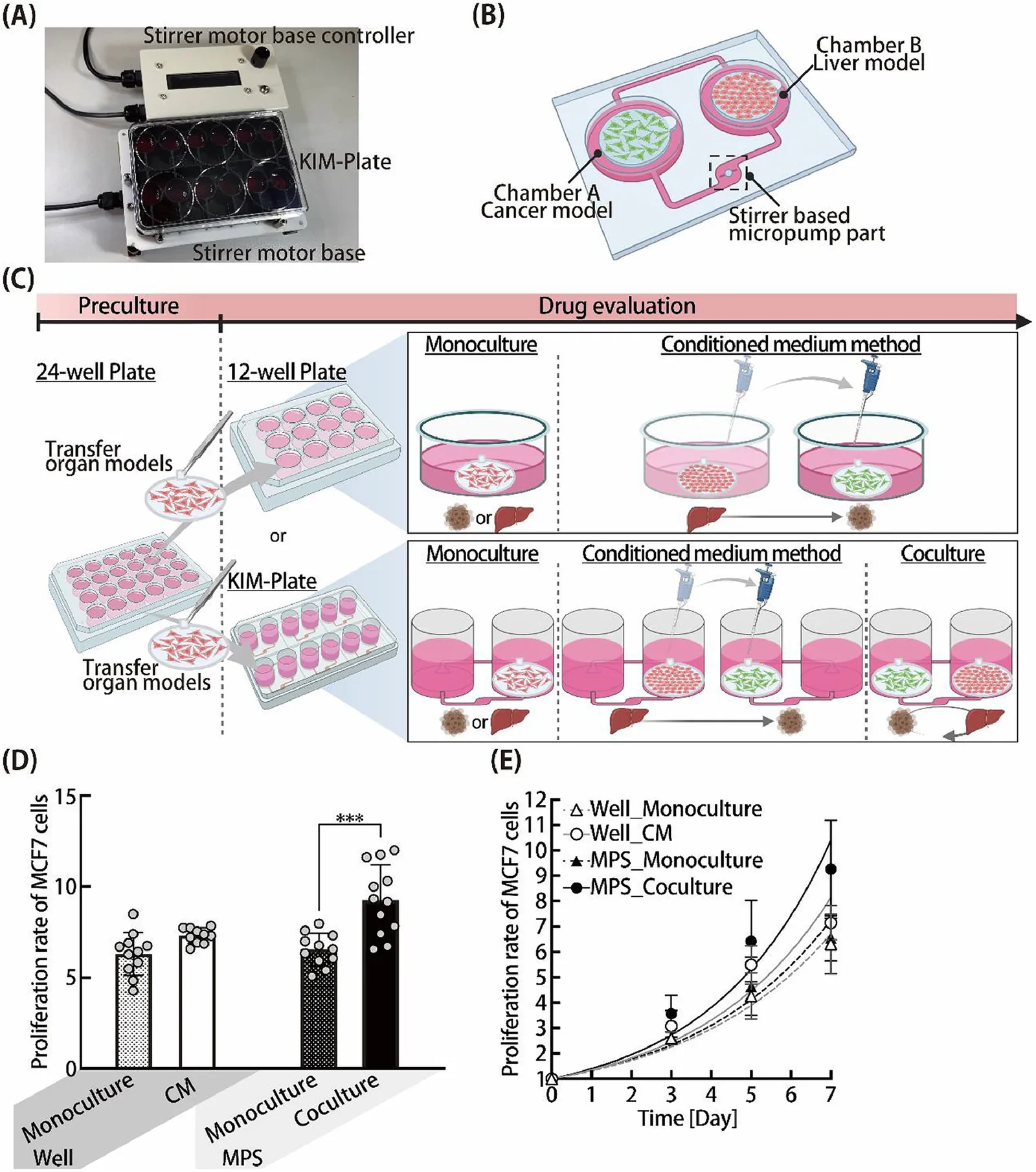
Kinetic-pump integrated microfluidic plate (KIM-Plate)-based liver-cancer microphysiological system (MPS). (A) Photographs of the KIM-Plate, the stirrer motor base, and its controller. The rotational speed of the stirrer motor controlled the flow rate of the KIM-Plate. (B) Schematic diagram of a multi-organ MPS unit (MO-MPS unit). PXB cells, as a liver model, and MCF7 cells, as a cancer model, were integrated into the culture chambers. (C) Schematic of culture methods using the KIM-Plate and well plates. (D) Proliferation rate of MCF7 cells at day 7 in each culture condition. (E) Proliferation rate of MCF7 cells by culture days. Each value was normalized to the CCK-8-derived signal measured on day 0. Error bars represent the standard deviation (SD), *** *p* < 0.001.

To evaluate whether simultaneous liver–cancer coculture affects breast cancer cell proliferation, PXB cells were used as the liver model and MCF7 cells as the cancer model (Fig. 1A and B). Four culture conditions were compared: well-plate monoculture (Well_Monoculture), a conventional conditioned medium method using well plates (Well_CM), KIM-Plate monoculture (MPS_Monoculture), and simultaneous coculture using the KIM-Plate (MPS_Coculture) (Fig. 1C). In the MPS_Coculture condition, PXB and MCF7 cells were cultured in separate chambers connected by continuously circulating medium. In the Well_CM condition, culture supernatants collected from PXB cells were transferred intermittently to MCF7 cells. MCF7 cell proliferation was evaluated after 7 days of culture. Detailed culture procedures and operating conditions are described in Section 3.1 and Fig. S1.

After 7 days of MCF7 culture, no significant difference in the MCF7 cell proliferation rate was observed between the Well_Monoculture and Well_CM conditions (Fig. 1D). In contrast, the proliferation rate of MCF7 cells under the MPS_Coculture condition was significantly higher than that under the MPS_Monoculture condition. The estimated doubling times of MCF7 cells were 2.55 days (95% confidence interval [CI], 2.42–2.62 days) under the Well_Monoculture condition, 2.31 days (95% CI, 2.20–2.38 days) under the Well_CM condition, 2.45 days (95% CI, 2.30–2.54 days) under the MPS_Monoculture condition, and 2.07 days (95% CI, 1.95–2.13 days) under the MPS_Coculture condition (Fig. 1E). Thus, the estimated doubling time was numerically shorter under conditions involving PXB cells than under the corresponding monoculture conditions, although a statistically significant increase in MCF7 cell proliferation relative to the corresponding monoculture was observed only under the MPS_Coculture condition.

Hepatocyte growth factor (HGF)- and insulin-like growth factor (IGF)-related signaling pathways have been reported to promote the proliferation of various cancer cells, including breast cancer cells (Ziegler et al., 2016; Owusu et al., 2017; Zhang et al., 2011). Cancer cell proliferation can also be regulated through the combined actions of multiple soluble factors. For example, estradiol potentiates the growth-promoting effects of IGF, epidermal growth factor, transforming growth factor-α, and basic fibroblast growth factor on MCF7 cells (Stewart et al., 1992; Dupont and Le Roith, 2001). Moreover, fibroblasts and adipocytes cocultured with breast cancer cells can acquire secretory phenotypes associated with breast cancer proliferation and metastasis (Tyan et al., 2011; Wang et al., 2015). These findings support the broader possibility that cancer cells can alter the behavior and secretory profiles of neighboring cells, although such changes were not directly examined in the present study.

The enhanced proliferation of MCF7 cells observed under conditions involving PXB cells might therefore have been mediated by soluble factors. The difference between the MPS_Coculture and Well_CM conditions may be attributable to differences in the directionality and timing of intercellular communication. In the Well_CM condition, soluble factors produced during PXB cell culture were transferred intermittently and could act unidirectionally on MCF7 cells. In contrast, in the MPS_Coculture condition, PXB and MCF7 cells shared a continuously circulating medium. Consequently, PXB cells were also exposed to factors released by MCF7 cells, potentially allowing bidirectional communication between the two cell types. Furthermore, soluble factor secretion and MCF7 cell exposure occurred continuously in the MPS_Coculture condition, whereas the Well_CM condition involved a delay between factor secretion and exposure. In addition, the MPS_Coculture condition involved perfusion, which may have enhanced fluid mixing and mass transfer during exposure to soluble factors released by PXB cells. In the Well_CM condition, MCF7 cells were cultured using conditioned medium collected after PXB cell culture; therefore, the culture environment at the beginning of MCF7 culture differed from that in the MPS_Coculture condition, including the availability of nutrients and other soluble components in the medium. Thus, various differences between the two conditions, including the timing and continuity of exposure to soluble factors, the presence or absence of perfusion, and the concentrations of nutrients and other soluble components available to MCF7 cells at the beginning of culture, may have contributed to the differences observed in MCF7 proliferation. By contrast, the initial numbers of PXB and MCF7 cells and the culture medium volume were matched between the corresponding conditions (3.1, 3.2); therefore, differences in initial cell density are unlikely to account for the observed difference in MCF7 proliferation.

Therefore, the present results demonstrate that dynamic liver–cancer cell coculture under perfusion is associated with enhanced MCF7 proliferation compared with the MPS_Monoculture condition, but do not establish the molecular mechanisms responsible for this phenotype. To determine whether specific intercellular signals contribute to the observed phenotype, direct measurement of soluble factors, together with analysis of cellular responses in both PXB and MCF7 cells, would be required. Nevertheless, when the proliferation rate on day 7 was compared within each culture format, a statistically significant increase associated with the presence of PXB cells was observed only under the simultaneous coculture condition: no significant difference was found between Well_Monoculture and Well_CM, whereas MPS_Coculture differed significantly from MPS_Monoculture. Because each of these comparisons was performed under a matched culture format and matched perfusion status, this contrast indicates that the KIM-Plate can capture a proliferative response that was not detected using the conventional CM transfer method. It should be noted, however, that the estimated doubling times were numerically shorter in both the CM and coculture conditions than in the corresponding monocultures, and that the present design does not allow the perfused environment and the simultaneous presence of PXB cells to be separated when Well_CM and MPS_Coculture are compared directly. Independently of these considerations, MCF7 cell proliferation could be quantified separately under all culture conditions, confirming that the established liver–cancer MPS provided a suitable platform for the subsequent anticancer drug efficacy experiments.

### 2.2 CPT-11 metabolism by PXB cells under static and perfusion conditions

To characterize CPT-11 metabolism under the conditions used in the subsequent drug efficacy experiments, PXB cells were cultured alone and exposed to 1,000 nM CPT-11 for 72 h. CPT-11 metabolism was compared under static and perfusion conditions using a 12-well plate and the KIM-Plate, respectively. The culture medium was collected every 24 h, and the concentrations of CPT-11 and its metabolites, SN-38 and SN-38 glucuronide (SN-38G), were quantified using HPLC–MS/MS.

**Fig. 2:**
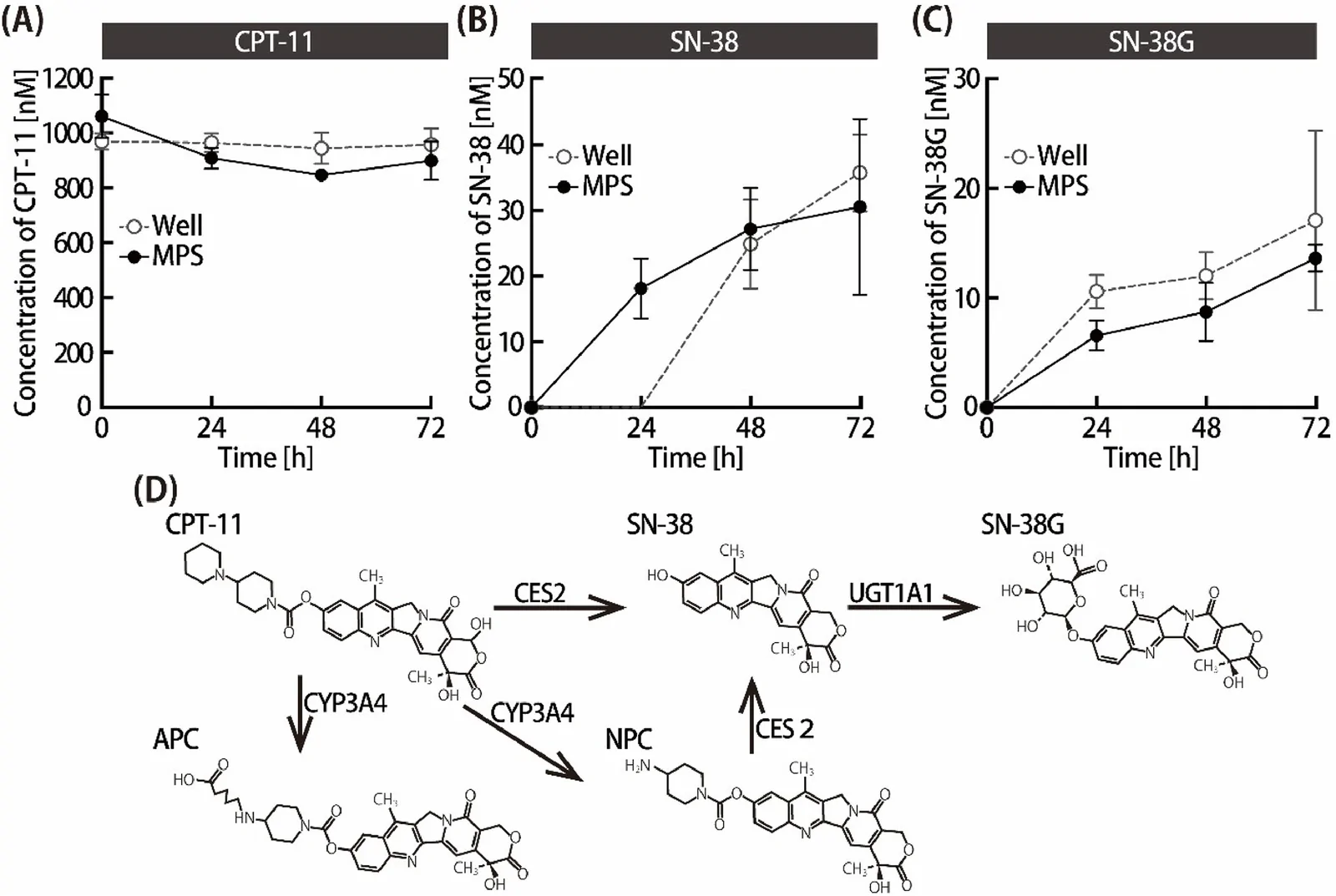
Metabolism of CPT-11 by PXB cells. (A) Concentration changes of CPT-11. (B) Concentration changes of SN-38, a product of phase I metabolism. (C) Concentration changes of SN-38G, a product of phase II metabolism. Error bars represent the standard deviation (SD). (D) Metabolic pathway of CPT-11: CPT-11 is metabolized to SN-38, with anticancer effect, by CES2, and to APC and NPC, without anticancer effect, by CYP3A4. NPC is metabolized to SN-38 by CES2, and SN-38 is converted into its inactive metabolite, SN-38G, via UGT1A1-mediated glucuronidation.

The CPT-11 concentration remained largely unchanged over 72 h under both culture conditions (Fig. 2A). The SN-38 concentration increased substantially during the first 48 h, followed by only a limited increase from 48 to 72 h (Fig. 2B). In contrast, the SN-38G concentration increased gradually throughout the 72-h culture period under both conditions (Fig. 2C). CPT-11, SN-38, and SN-38G exhibited similar concentration–time profiles in the well plate and KIM-Plate. At 72 h, no significant differences between the two culture conditions were observed in the concentrations of CPT-11, SN-38, or SN-38G.

CPT-11 is a prodrug used to treat several types of cancer, including colorectal, breast, lung, and ovarian cancers. Its active metabolite SN-38 exerts potent anticancer activity by inhibiting topoisomerase I, an enzyme required for DNA replication (Kurita and Kaneda, 1999). CPT-11 is hydrolyzed mainly by carboxylesterase 2 (CES2) to form SN-38, which exhibits substantially greater anticancer activity than CPT-11 (Uchida et al., 2013). CPT-11 is also metabolized by CYP3A4 to form the oxidative metabolites 7-ethyl-10-[4-N-(5-aminopentanoic acid)-1-piperidino]carbonyloxycamptothecin (APC) and 7-ethyl-10-[4-(1-piperidino)-1-amino]carbonyloxycamptothecin (NPC) (Fig. 2D) (Uchida et al., 2013; Mullangi et al., 2010; Sanghani et al., 2004). NPC can subsequently be converted to SN-38 by CES2, whereas SN-38 is glucuronidated by UDP-glucuronosyltransferase 1A1 (UGT1A1) to form the inactive metabolite SN-38G.

The production of both SN-38 and SN-38G confirmed that PXB cells retained the capacity for sequential metabolism of CPT-11 to SN-38 and SN-38G under both static and perfusion conditions. The amount of SN-38 detected was smaller than would be expected from the apparent decrease in CPT-11 alone. This discrepancy may reflect the formation of unmeasured metabolites, including APC and NPC, as well as other processes such as compound degradation, adsorption, or sampling-associated loss.

The similar concentration–time profiles observed in the well plate and KIM-Plate suggest that perfusion culture under the conditions used in this study did not substantially alter the measured CPT-11 metabolic profile of PXB cells. These results support the use of the KIM-Plate in subsequent liver–cancer coculture experiments without an apparent loss of the measured CPT-11-metabolizing activity of PXB cells. In addition, the open culture chambers of the KIM-Plate enabled repeated medium sampling and direct measurement of drug and metabolite concentrations using procedures comparable to those used for conventional well plates. The metabolic profiles obtained in this experiment were subsequently used to evaluate the effects of time-dependent CPT-11 metabolism on cancer cell responses in the liver–cancer coculture system.

### 2.3 Drug efficacy testing of SN-38 and CPT-11 in monoculture of MCF7 cells

The responses of MCF7 cells to CPT-11 and SN-38 were evaluated under static and perfusion conditions to establish concentration–response relationships for the subsequent analysis of metabolism-dependent drug exposure. MCF7 cells were cultured for 7 days in either a 12-well plate under static conditions or the KIM-Plate under perfusion conditions. The cells were exposed to CPT-11 or SN-38 from days 0 to 3 and subsequently cultured in drug-free medium from days 3 to 7. Drug responses were evaluated based on MCF7 cell proliferation and normalized cell viability.

**Fig. 3:**
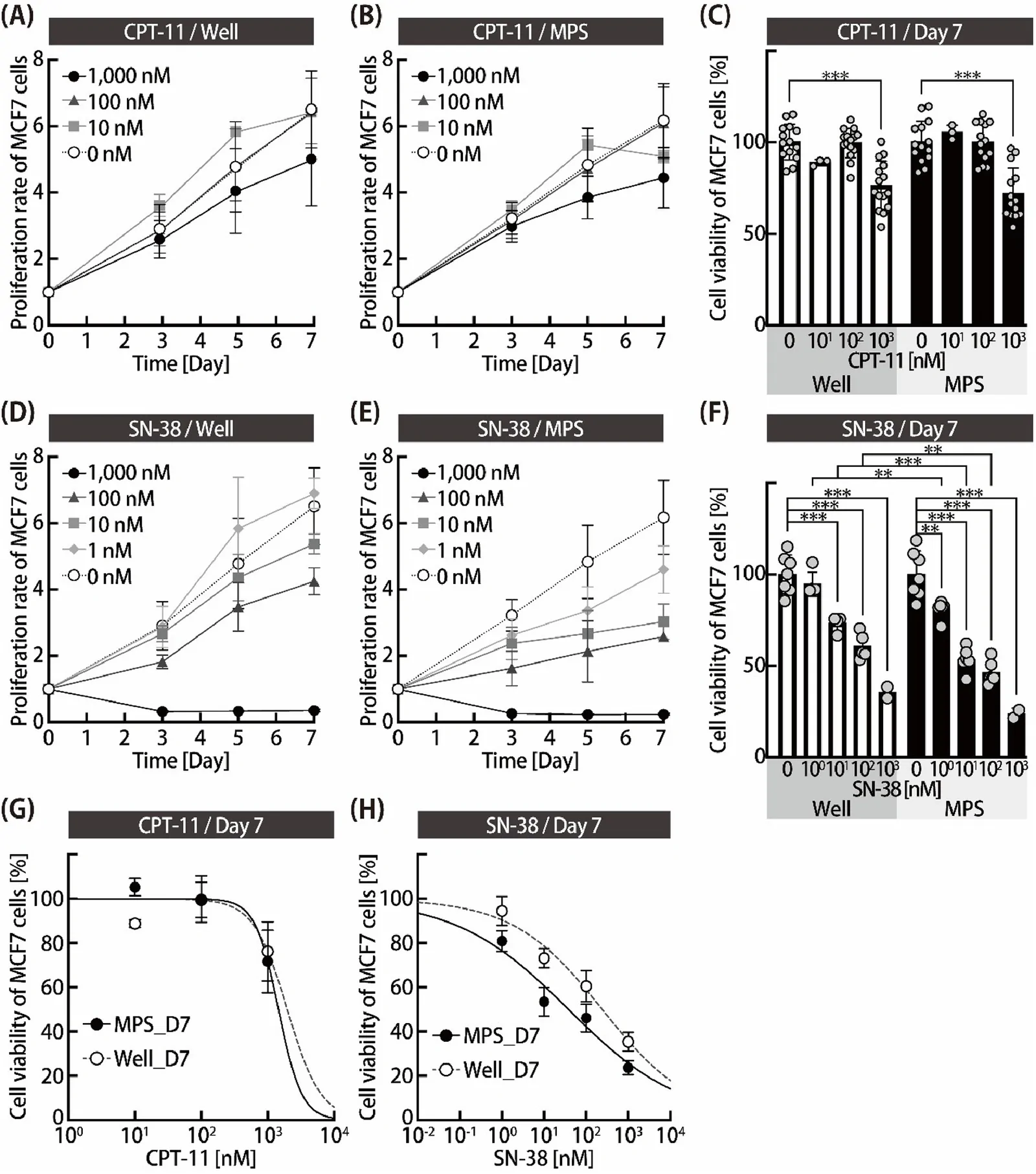
. Drug efficacy of CPT-11 and SN-38 in MCF7 monocultures. Cell proliferation rate of MCF7 cells (A) exposed to CPT-11 in a well plate and (B) exposed to CPT-11 in the MPS. Each value was normalized to the CCK-8-derived signal measured on day 0. (C) Cell viability of MCF7 cells on day 7. Cell proliferation rate of MCF7 cells (D) exposed to SN-38 in a well plate and (E) exposed to SN-38 in the MPS. Each value was normalized to the CCK-8-derived signal measured on day 0. (F) Cell viability of MCF7 cells on day 7. (G) CPT-11 and (H) SN-38 concentration-cell viability curves used to calculate 50% growth inhibition (GI50). Error bars represent the standard deviation (SD), ** *p* < 0.01, *** *p* < 0.001.

Exposure to 1,000 nM CPT-11 reduced the proliferation rate of MCF7 cells relative to the vehicle control under both static and perfusion conditions, and the difference between the control and CPT-11-exposed groups increased over time (Fig. 3A and B). On day 7, MCF7 cell viability did not differ significantly from that of the control at CPT-11 concentrations of ≤100 nM but was significantly reduced at 1,000 nM under both conditions (Fig. 3C). No significant differences in cell viability were observed between the well-plate and KIM-Plate conditions at any CPT-11 concentration tested.

In contrast, SN-38 reduced MCF7 cell proliferation in a concentration-dependent manner under both static and perfusion conditions (Fig. 3D and E). At 1,000 nM SN-38, the proliferation rate decreased to below 1 under both conditions, suggesting that the relative number of viable MCF7 cells was lower than that at the beginning of drug exposure. On day 7, cell viability in the well plate was comparable to that of the control at 1 nM SN-38 but was significantly reduced at 10 and 100 nM (Fig. 3F). Under perfusion conditions in the KIM-Plate, cell viability was significantly reduced even at 1 nM SN-38. Moreover, cell viability was significantly lower in the KIM-Plate than in the well plate at SN-38 concentrations ranging from 1 to 100 nM.

CPT-11 did not reduce MCF7 cell growth by 50% even at the highest concentration tested; therefore, its 50% growth inhibitory concentration (GI50) could not be determined within the tested concentration range (Fig. 3G). In contrast, the GI50 values of SN-38 were 242.2 nM (95% confidence interval [CI], 91.02–439.7 nM) in the well plate and 38.2 nM (95% CI, 12.3–66.5 nM) in the KIM-Plate (Fig. 3H). Thus, the GI50 of SN-38 under perfusion conditions was approximately 6.3-fold lower than that under static conditions, indicating greater apparent sensitivity of MCF7 cells to SN-38 in the KIM-Plate.

The substantially greater potency of SN-38 than CPT-11 is consistent with the established pharmacological properties of these compounds (Uchida et al., 2013). Growth inhibition by CPT-11 was detected only at 1,000 nM, whereas SN-38 produced measurable effects at concentrations two to three orders of magnitude lower. The plasma maximum concentration (Cmax) of SN-38 in patients receiving 125 mg/m² CPT-11 has been reported to be 12.9 ± 5.5 ng/mL, corresponding to approximately 32.9 nM (Younis et al., 2009). The GI50 obtained in the KIM-Plate was therefore of the same order of magnitude as the reported plasma Cmax. However, this comparison is limited to the order of magnitude of the concentration and does not establish clinical relevance, because the duration and temporal profile of SN-38 exposure in the present experiment differed from those observed clinically and because plasma protein binding and tissue distribution were not represented in the present system.

One possible explanation for the lower GI50 under perfusion conditions is that medium circulation reduced local mass-transfer limitations and maintained a more uniform drug concentration around the cells. Our previous fluid simulation indicated that the shear stress applied to the cell culture area of the KIM-Plate was low (Yap et al., 2025), suggesting that the difference in drug response was unlikely to be attributable primarily to direct mechanical stimulation. In addition, our previous study demonstrated that perfusion in the KIM-Plate enhanced drug-induced hepatotoxic responses, potentially by facilitating molecular transport around the cultured cells (Shinha et al., 2025). Although drug concentration gradients were not directly measured in the present study, these findings suggest that perfusion-dependent mass transport contributed to the greater apparent sensitivity of MCF7 cells to SN-38. Thus, the KIM-Plate provides a culture environment in which the effects of perfusion on drug responses can be evaluated while retaining compatibility with conventional well-plate-based assays.

This observation also has an implication for the interpretation of the liver–cancer coculture experiments described below. Because the perfused environment itself altered the apparent potency of SN-38, differences between conventional static assays and the KIM-Plate liver–cancer coculture reflect both fluidic transport and the integration of the hepatic and cancer compartments. For this reason, a perfused conditioned-medium condition (MPS_CM) was included in the following experiments so that the effect associated with the simultaneous presence of hepatocytes could be evaluated under matched perfusion conditions.

### 2.4 Drug efficacy testing of CPT-11 considering hepatic metabolism

The response of MCF7 cells to CPT-11 in the presence of hepatic metabolism was evaluated under three conditions: simultaneous coculture of PXB and MCF7 cells in the KIM-Plate (MPS_Coculture), exposure of MCF7 cells in the KIM-Plate to conditioned medium generated by PXB cells (MPS_CM), and exposure of MCF7 cells in a 12-well plate to PXB cell-conditioned medium (Well_CM) (Fig. S1). The three conditions were designed to distinguish the effects associated with the perfused MPS environment from those associated with simultaneous liver–cancer coculture (Table S1). Comparison of the Well_CM and MPS_CM conditions allowed the influence of the culture environment, including perfusion-dependent effects, to be evaluated using PXB cell-conditioned medium. In contrast, both MPS_CM and MPS_Coculture were performed in the KIM-Plate under the same perfusion conditions; therefore, comparison of these two conditions was used to evaluate the effect associated with the continuous presence of PXB cells during drug exposure of MCF7 cells. In all conditions, cells were exposed to drug-containing medium for the first 3 days and subsequently cultured in drug-free medium for 4 days. Drug responses were evaluated based on normalized MCF7 cell viability on day 7. In addition, cell viability under each condition was estimated using the CPT-11 metabolic profiles obtained from PXB monocultures and the concentration–response relationships obtained from MCF7 monocultures.

**Fig. 4:**
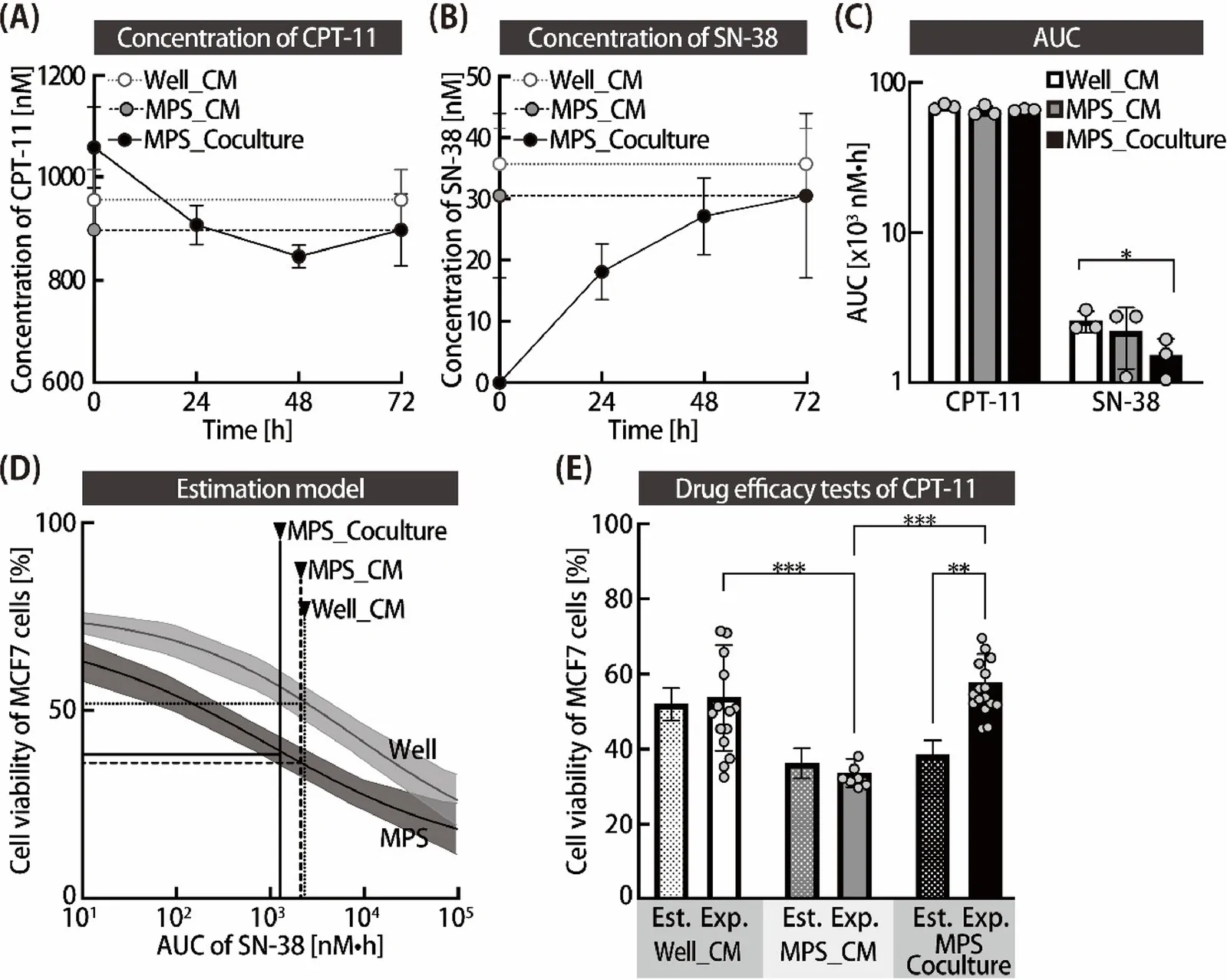
Estimation and experimental results of CPT-11 efficacy using the conditioned medium (CM) and MPS_Coculture conditions. Estimated concentration-time curves of (A) CPT-11 and (B) SN-38. (C) Estimated AUCs of CPT-11 and SN-38 in each condition. (D) AUC-cell viability curve used to predict drug efficacy for each condition. Colored areas indicate 95% confidence intervals. (E) Drug efficacy of CPT-11 in the Well_CM, MPS_CM, and MPS_Coculture conditions. Error bars represent the standard deviation (SD), * *p* < 0.05, ** *p* < 0.01, *** *p* < 0.001.

The CPT-11 and SN-38 exposure profiles during the 72-h drug-exposure period were estimated from the metabolic profiles obtained in PXB monoculture experiments. These model-derived profiles were used to calculate the areas under the concentration–time curves (AUCs) for each condition. In the MPS_Coculture condition, the estimated concentrations changed over time because CPT-11 was continuously metabolized by PXB cells. In the CM conditions, MCF7 cells were exposed to PXB-conditioned medium, and the concentrations present at the beginning of exposure were assumed to remain constant during the exposure period (Fig. 4A and B). The CPT-11 AUC did not differ significantly among the three conditions (Fig. 4C). In contrast, the SN-38 AUC in the MPS_Coculture condition was significantly lower and was approximately 60% of that in the Well_CM condition. No significant difference in SN-38 AUC was observed between the Well_CM and MPS_CM conditions.

The expected MCF7 cell viability under each condition was estimated from the drug-exposure data obtained in the monoculture experiments. Because CPT-11 produced similar effects under static and perfusion conditions in MCF7 monocultures (Fig. 3C), its direct contribution was estimated using the viability measured following exposure to 1,000 nM CPT-11. The contribution of SN-38 was estimated using the relationship between SN-38 AUC and MCF7 cell viability (Fig. 4D). Based on this model, the estimated cell viability values were 52% (95% confidence interval [CI], 47–56%) for Well_CM, 36% (95% CI, 32–40%) for MPS_CM, and 38% (95% CI, 34–40%) for MPS_Coculture.

Experimentally, the normalized viability of MCF7 cells was significantly lower under the MPS_CM condition (33%) than under the Well_CM (53%) and MPS_Coculture (57%) conditions (Fig. 4E). Comparable responses were observed between the Well_CM and MPS_Coculture conditions over the tested CPT-11 concentration range of 10–1,000 nM (Fig. S3). The experimentally observed viability values were similar to the estimated values under both the Well_CM and MPS_CM conditions. In contrast, the experimentally observed viability under the MPS_Coculture condition was substantially higher than the estimated value.

Although the Well_CM and MPS_CM conditions had comparable estimated SN-38 AUCs, cell viability was lower under the MPS_CM condition. This difference is consistent with the greater apparent sensitivity to SN-38 observed under perfusion conditions in the MCF7 monoculture experiment (Fig. 3F and H), suggesting that perfusion-dependent mass transport contributed to the enhanced drug response. In contrast, the MPS_CM and MPS_Coculture conditions were both performed under the same perfusion conditions. The greater response observed under the MPS_CM condition than under the MPS_Coculture condition could be partly explained by the higher estimated SN-38 AUC under the MPS_CM condition. The higher cumulative SN-38 exposure was associated with lower MCF7 cell viability. However, AUC alone did not fully explain the observed difference.

The discrepancy between the estimated and experimentally observed viability under the MPS_Coculture condition suggested that the temporal profile of SN-38 exposure may also have contributed to the response of MCF7 cells. In the MPS_Coculture condition, the estimated SN-38 concentration gradually increased as CPT-11 was metabolized by PXB cells. In contrast, the CM conditions exposed MCF7 cells to SN-38 that was already present in the conditioned medium at the beginning of exposure. To examine whether this difference in exposure profile affected the drug response independently of total exposure, MCF7 cells were exposed to either constant or increasing SN-38 concentrations at similar AUCs (Supplemental Experiment 1 and Fig. S4). Cell viability was higher under the increasing-concentration conditions than under the constant-concentration conditions. The AUC-based GI50 was 192.5 nM·h under the constant-concentration condition and 633.4 nM·h under the increasing-concentration condition, corresponding to an approximately 3.3-fold difference.

Low-concentration drug exposure may also have contributed to the response observed under the MPS_Coculture condition. In the MCF7 monoculture experiments, cells exposed to 10 nM CPT-11 or 1 nM SN-38 showed slightly higher proliferation rates than the corresponding unexposed controls (Fig. 3A and D). Although the biological basis and statistical robustness of these increases require further investigation, the results raise the possibility that initial exposure to low SN-38 concentrations altered the subsequent response to increasing drug concentrations.

A coculture-associated change in the cellular state of MCF7 cells may also have contributed to the observed discrepancy. The presence of PXB cells altered MCF7 cell proliferation under drug-free conditions (Section 2.1), and such a change in cellular state cannot be excluded under drug exposure. Because the normalized viability was calculated relative to a drug-free control cultured under the same condition, differences in the baseline proliferation rate were accounted for. However, the concentration–response relationships used for the estimation were obtained in MCF7 monocultures, and a shift in these relationships under coculture conditions, as well as differences in the effective intracellular exposure to SN-38, may also have contributed to the discrepancy between the estimated and observed responses.

Taken together, the lower-than-expected response observed under the MPS_Coculture condition may have reflected both a lower cumulative exposure to SN-38 and an exposure-profile-dependent decrease in apparent drug sensitivity. The present experimental design does not allow the individual contributions of hepatic metabolism, perfusion, perfusion-dependent mass transfer, the continuity of drug and soluble-factor exposure, and direct or indirect intercellular communication to be separated. Each of these factors may have contributed to the response observed under the MPS_Coculture condition. Nevertheless, the results support the interpretation that the response of MCF7 cells cannot be fully predicted from the AUC of SN-38 alone and may depend on the temporal pattern of metabolite exposure. By continuously generating active metabolites through hepatic metabolism, the KIM-Plate liver–cancer coculture provides an experimental system for investigating metabolism-dependent drug responses that are difficult to reproduce using conventional conditioned medium transfer methods.

### 2.5 Limitations

Several limitations of the present study should be considered. First, only one combination of a cancer cell line and an anticancer drug was examined. Although the GI_50_ of SN-38 obtained in the KIM-Plate was of the same order of magnitude as a previously reported clinical plasma C_max_, this comparison was limited to the order of magnitude of the concentration and does not establish clinical relevance. The duration and temporal profile of drug exposure in the present experiments differed from those observed clinically, and plasma protein binding and tissue distribution were not represented in the present system. Therefore, the general applicability of the platform should be evaluated using additional cancer models, anticancer drugs, and clinically relevant exposure profiles.

Second, the liver–cancer MPS used in this study did not fully recapitulate the pharmacokinetic processes that occur in the human body. The system lacked drug excretion and other organ-level disposition processes, and the culture medium volume was large relative to the number of hepatocytes. As a simplified scaling estimate, assuming approximately 2.1 × 10¹¹ hepatocytes in the human liver (Pek et al., 2021), a blood volume of 5,600 mL, and drug distribution limited to the blood compartment, the medium volume per hepatocyte in the present MPS was approximately 140.6-fold greater than the corresponding blood volume per hepatocyte in vivo. This simplified calculation does not account for tissue distribution, plasma protein binding, or differences in metabolic activity between cultured PXB cells and hepatocytes in vivo. Nevertheless, it indicates that the hepatic metabolic capacity relative to the fluid volume was substantially lower in the present system than in the human body. Consequently, metabolism-dependent changes in CPT-11 and SN-38 concentrations were less pronounced than would be expected under physiological conditions.

In addition, drug and metabolite concentrations were not measured directly during the liver–cancer coculture efficacy experiments. In the metabolic characterization experiments described in Section 2.2, repeated medium sampling was acceptable because no pharmacodynamic endpoint was evaluated in parallel. In the efficacy experiments, however, repeated sampling would have reduced the amounts of drug and metabolites in the circulating medium and would thereby have altered the drug exposure to which the MCF7 cells were subjected. Because this exposure is the variable under investigation, preserving it was prioritized over obtaining concentration measurements.

Therefore, the drug-exposure profiles used to estimate MCF7 cell responses were derived from metabolic data obtained in PXB monocultures, and possible effects of MCF7 cells and coculture-dependent interactions on drug disposition were not incorporated into the analysis. The presence of MCF7 cells may have influenced the actual drug and metabolite concentrations during coculture. MCF7 cells can take up and retain CPT-11 and SN-38 intracellularly, and a contribution of MCF7 cells themselves to drug disposition cannot be excluded. However, such contributions are expected to be limited relative to those of the PXB cells, because the number of MCF7 cells introduced was more than an order of magnitude lower than that of PXB cells (Section 3.2), and because CPT-11 metabolism is mediated mainly by hepatic enzymes. The estimated exposure profiles used in this analysis therefore represent the drug and metabolite concentrations generated by hepatic metabolism, and direct measurement during coculture will be required to determine the extent to which the presence of MCF7 cells modifies these profiles.

Future studies should improve physiological scaling by increasing the hepatocyte-to-medium ratio, for example through the use of hepatocyte spheroids or a reduction in circulating medium volume. Incorporation of additional disposition functions, including drug excretion, and direct measurement of drug and metabolite concentrations during coculture would further improve the prediction of metabolism-dependent drug responses. Validation using multiple cancer cell–drug combinations will also be required to establish the broader applicability of the KIM-Plate for pharmacodynamic studies.

## 3 Materials and methods

### 3.1 Kinetic-pump integrated microfluidic plate and culture conditions for liver–cancer interaction experiments

A liver–cancer MPS was constructed using the Kinetic-pump integrated microfluidic plate (KIM-Plate), which was previously developed by our group (Shinha et al., 2021). The KIM-Plate contained six independent multi-organ MPS units within an ANSI/SLAS-standard microplate footprint. Each unit comprised two open culture chambers connected by microchannels to form a recirculating flow path. Culture medium was circulated between the chambers using a stirrer-based on-chip pump. The culture chambers were comparable in size to the wells of a conventional 24-well plate and accommodated Cell Desks carrying the cultured cells. Cells used in the following experiments were introduced into each culture system on Cell Desks seeded at identical densities during the preculture period (Section 3.2). Therefore, the initial numbers of PXB and MCF7 cells were identical between the corresponding conditions described below.

For the MPS_Coculture condition, PXB and MCF7 cells precultured on Cell Desks were transferred to separate chambers of the KIM-Plate on day 0 and cocultured for 7 days. DMSO-free dHCGM was used as the common culture medium. Each MPS unit contained 1,500 µL of medium. The medium was changed on days 3 and 5. The rotational speed of the stirrer-based pump was set to 6,500 rpm, corresponding to an approximate flow rate of 36 µL/min. The cultures were maintained at 36.5°C, 5% CO₂, and ≥95% relative humidity to maintain the culture medium at approximately 37°C. For the MPS_Monoculture condition in the liver–cancer interaction experiment, PXB or MCF7 cells were cultured individually in the KIM-Plate under the same medium, medium volume, pump, and incubation conditions as those used for the MPS_Coculture condition.

For the Well_CM condition, each well contained 1,500 µL of DMSO-free dHCGM, matching the medium volume of the MPS_Coculture condition. PXB cells were transferred to a 12-well plate on day 0. MCF7 cells were transferred to a separate 12-well plate on day 3 and cultured for 7 days. PXB cell culture supernatant collected on day 3 was transferred directly to the MCF7 culture. Supernatants collected from PXB cells on days 5 and 7 were stored at 4°C and used for MCF7 medium changes on days 6 and 8, respectively (Fig. S1). Well-plate monocultures were performed in parallel. All well-plate cultures were maintained at 37°C with 5% CO₂ in a humidified atmosphere.

### 3.2 Cell preparation

PXB cells (PhoenixBio Co., Ltd., Hiroshima, Japan) were used as the liver model, and MCF7 cells (JCRB0134; JCRB Cell Bank, Osaka, Japan) were used as the breast cancer model. Both cell types were seeded onto Cell Desks (MS-0113K; Sumitomo Bakelite Co., Ltd., Tokyo, Japan) coated with collagen (IAC-30; KOKEN Co., Ltd., Tokyo, Japan) and precultured in conventional 24-well plates. All precultures were maintained at 37°C in a humidified atmosphere containing 5% CO₂.

The PXB cells used as the liver model are fresh human hepatocytes isolated from chimeric mice with humanized livers and have been reported to retain human hepatocyte characteristics and drug-metabolizing activities during long-term culture (Yamasaki et al., 2020). PXB cells were seeded onto the Cell Desks at a density of 2.1 × 10⁵ cells/cm² at least 1 week before the start of each experiment. The cells were maintained in dHCGM (PhoenixBio Co., Ltd.), as recommended by the manufacturer, and the medium was changed 3-4 days during the preculture period.

MCF7 cells were seeded onto the Cell Desks at a density of 1.5 × 10⁴ cells/cm² 2 days before the start of each experiment. The MCF7 culture medium consisted of Minimum Essential Medium Eagle (MEME; M5650-500ML, Merck KGaA, Darmstadt, Germany) supplemented with 10% fetal bovine serum (FBS; 10270106, Gibco, MA, USA), 0.01 mg/mL insulin (I9278-5ML; Merck KGaA), and 1% antibiotic–antimycotic solution (161-23181; FUJIFILM Wako Pure Chemical Corporation, Osaka, Japan).

At the start of each experiment, the Cell Desks carrying PXB or MCF7 cells were transferred to the designated KIM-Plate or well-plate culture systems, as described in Section 3.1.

### 3.3 Calculation of the cell proliferation rate and viability

The relative number of viable MCF7 cells was evaluated using a Cell Counting Kit-8 (CCK-8; Dojindo Molecular Technologies, Inc., Kumamoto, Japan). The CCK-8 assay is based on the reduction of WST-8 by cellular dehydrogenase activity through an electron mediator, resulting in the formation of a water-soluble formazan dye. Within the appropriate assay range, the amount of formazan produced correlates with the number of viable cells. Therefore, the CCK-8-derived signal was used in this study as an indicator of the relative number of viable MCF7 cells rather than as a direct measurement of absolute cell number. At the specified measurement time points, the Cell Desks carrying MCF7 cells were transferred to fresh 24-well plates before the assay. This procedure enabled MCF7-specific measurements without interference from PXB cells cultured in the other chamber of the KIM-Plate. The *proliferation rate* was calculated using Eq. (1):

(1) $$proliferation\;rate=\;\frac{S_{t}}{S_{0}}$$

where (*St*) is the CCK-8-derived signal at the indicated time point and (*S0*) is the signal measured on day 0.

Normalized cell viability was calculated relative to the corresponding drug-unexposed control using Eq. (2):

(2) $$Nomalized\;cell\;viability=\frac{S_{drug}}{S_{control}}\times 100$$

where (*Sdrug*) is the CCK-8-derived signal in the drug-exposed group and (*Scontrol*) is the signal in the drug-unexposed control.

The drug-unexposed control was cultured under the same platform and culture conditions as the corresponding drug-exposed group.

### 3.4 Calculation of the 50% growth inhibition (GI50)

The relationship between normalized MCF7 cell viability and drug concentration or SN-38 area under the concentration–time curve (AUC) was analyzed by nonlinear regression using GraphPad Prism version 10.3.0 (GraphPad Software, Boston, MA, USA). Normalized cell viability was expressed as a fraction relative to the corresponding drug-unexposed control. The upper plateau was fixed at 1, and the data were fitted using Eq. (3):

(3) $$y=Bottom+\frac{1-Bottom}{{\left(\frac{GI_{50}}{X}\right)}^{HillSlope}+1}$$

where *y* is the normalized cell viability, *x* is the drug concentration or SN-38 AUC, Bottom is the fitted lower plateau, GI50 is the value of *x* producing a response halfway between the upper and lower plateaus, and HillSlope is the slope factor describing the steepness of the concentration–response or AUC–response curve. The fitted GI50 value was used as an indicator of relative drug potency.

### 3.5 Calculation of the cell doubling time

The doubling time of MCF7 cells was estimated from the proliferation indices measured during the culture period. The relationship between the proliferation rate and culture time was fitted by nonlinear regression using GraphPad Prism according to Eq. (4):

(4) $$Proliferation\;rate=e^{\left(k\times t\right)}$$

where *k* represents the rate constant of MCF7 cells and *t* is the culture time.

### 3.6 Quantification of drug concentration using High-Performance Liquid Chromatography-Tandem Mass Spectrometry (HPLC-MS/MS)

The CPT-11-metabolizing activity of PXB cells was evaluated using HPLC-MS/MS. Drug and metabolite concentrations were measured in the culture medium collected 0–72 h. The collected sample (20 µL) was mixed with 150 µL of methanol and 30 µL of water containing 1 µM of imipramine as an internal standard. After vortexing, the solutions were centrifuged at 15,000 rpm for 10 min at 4°C, and the resulting supernatants were injected into the HPLC-MS/MS system. The amount of each metabolite was determined using an LC-MS8050 triple quadrupole mass spectrometer (SHIMADZU CORPORATION, Kyoto, Japan) coupled to an LC-30A system (SHIMADZU CORPORATION). Chromatography was performed using a CAPCELL PAK C18 MG III column (ID 2.0 × 50 mm; Osaka Soda Co., Ltd., Osaka, Japan) at 50°C through step-gradient elution with a flow rate of 0.4 mL/min according to the following program: 0–

0.5 min, 90% A/10% B; 0.5–2.5 min, 90% A/10% B to 10% A/90% B; 2.5–3.2 min, 10%

A/90% B; 3.2–3.3 min, 10%

A/90% B to 90% A/10% B; 3.3–4.5 min, 90% A/10% B; (A, water containing 0.1% formic acid; B, acetonitrile containing 0.1% formic acid). The detected mass numbers and collision energy (CE) were 587.3 > 393.1 at –42 eV for CPT-11, 393.3 > 349.0 at –26 eV for SN-38, 569.3 > 393.1 at –28 eV for SN-38G, and 280.9 > 86.15 at –17 eV for imipramine as an internal standard.

### 3.7 Statistical analysis

Each independently seeded and cultured well or KIM-Plate unit was treated as an independent experimental replicate. The number of replicates (n) for each experimental condition is provided in Supplementary Table S2.

Potential outliers were assessed separately within each experimental group using the Smirnov–Grubbs test at *P* < 0.05. All values are presented as the mean ± standard deviation (SD). Statistical analyses were performed using GraphPad Prism 10.3.0 (GraphPad Software, Boston, MA, USA). Comparisons between two groups were performed using Welch’s t-test. For comparisons involving culture condition and drug concentration as two factors, two-way analysis of variance (ANOVA) followed by Tukey’s multiple comparisons test was used. Assumptions for two-way ANOVA were assessed using the D’Agostino–Pearson omnibus test together with visual inspection of Q–Q plots, and by visual inspection of residual plots for homoscedasticity. All datasets subjected to two-way ANOVA met these criteria. For comparisons among three or more groups involving a single factor, one-way ANOVA followed by Tukey’s multiple comparisons test was used. Statistical significance was defined as *P* < 0.05.

## Funding

This research was supported by the Japan Agency for Medical Research and Development under grant numbers JP22be1004101, JP22be1004201, and JP17be0304201 (to Y.S. and H.K.) and the Program for Grant-in-Aid for Scientific Research (B) 18H01849 and 24K01321 (to H.K.).

## Generative AI statement

The author(s) declared that generative AI was used in the creation of this manuscript. The authors used the generative artificial intelligence tool ChatGPT (OpenAI, version 5.6) for language editing and manuscript drafting support, and confirmed that all content was reviewed, verified, and approved by the authors.

## CRediT authorship contribution statement

**Kenta Shinha:** Writing – original draft, Visualization, Validation, Methodology, Investigation. **Hiroko Nakamura:** Resources, Investigation, Formal analysis. **Hiroshi Arakawa:** Visualization, Formal analysis. **Masaki Nishikawa:** Investigation, Formal analysis. **Yukio Kato:** Writing – review & editing, Investigation, Funding acquisition, Formal analysis. **Yasuyuki Sakai:** Writing – review & editing, Funding acquisition. **Hiroshi Kimura:** Writing – review & editing, Supervision, Project administration, Funding acquisition, Conceptualization.

## Declaration of competing interest

The authors declare that they have no known competing financial interests or personal relationships that could have appeared to influence the work reported in this paper.

## Data Availability

Data will be made available on request.

## References

[bib0014] Abaci H.E., Shuler M.L. (2015). Human-on-a-chip design strategies and principles for physiologically based pharmacokinetics/pharmacodynamics modeling. Integr. Biol. (U. K.).

[bib0011] Arakawa H., Sugiura S., Kawanishi T., Shin K., Toyoda H., Satoh T., Sakai Y., Kanamori T., Kato Y. (2020). Kinetic analysis of sequential metabolism of triazolam and its extrapolation to humans using an entero-hepatic two-organ microphysiological system. Lab. Chip..

[bib0003] Avila A.M., Bebenek I., Bonzo J.A., Bourcier T., Davis Bruno K.L., Carlson D.B., Dubinion J., Elayan I., Harrouk W., Lee S.L., Mendrick D.L., Merrill J.C., Peretz J., Place E., Saulnier M., Wange R.L., Yao J., Zhao D., Brown P.C. (2020). An FDA/CDER perspective on nonclinical testing strategies: classical toxicology approaches and new approach methodologies (NAMs). Regul. Toxicol. Pharmacol..

[bib0023] Dupont J., Le Roith D. (2001). Insulin-like growth factor 1 and oestradiol promote cell proliferation of MCF-7 breast cancer cells: new insights into their synergistic effects. Mol. Pathol..

[bib0015] Kimura H., Ikeda T., Nakayama H., Sakai Y., Fujii T. (2015). An on-chip small intestine–liver model for pharmacokinetic studies. J. Lab. Autom.

[bib0006] Kimura H., Sakai Y., Fujii T. (2018). Organ/body-on-a-chip based on microfluidic technology for drug discovery. Drug Metab. Pharmacokinet..

[bib0005] Kimura H., Nishikawa M., Kutsuzawa N., Tokito F., Kobayashi T., Kurniawan D.A., Shioda H., Cao W., Shinha K., Nakamura H., Doi K., Sakai Y. (2025). Advancements in Microphysiological systems: exploring organoids and organ-on-a-chip technologies in drug development -focus on pharmacokinetics related organs. Drug Metab. Pharmacokinet..

[bib0026] Kurita A., Kaneda N. (1999). High-performance liquid chromatographic method for the simultaneous determination of the camptothecin derivative irinotecan hydrochloride, CPT-11, and its metabolites SN-38 and SN-38 glucuronide in rat plasma with a fully automated on-line solid-phase extraction system, PROSPEKT. J. Chromatogr. B Biomed. Sci. Appl..

[bib0018] Kurniawan D.A., Leo S., Inamatsu M., Funaoka S., Aihara T., Aiko M., Rei I., Sakura T., Arakawa H., Kato Y., Matsugi T., Esashika K., Shiraki N., Kume S., Shinha K., Kimura H., Nishikawa M., Sakai Y. (2024). Gut–liver microphysiological systems revealed potential crosstalk mechanism modulating drug metabolism. PNAS. Nexus..

[bib0008] Lee S., Sung J. (2017). Microtechnology-based multi-organ models. Bioengineering.

[bib0013] Lee H., Kim D.S., Ha S.K., Choi I., Lee J.M., Sung J.H. (2017). A pumpless multi-organ-on-a-chip (MOC) combined with a pharmacokinetic–pharmacodynamic (PK–PD) model *Biotechnol*. Bioeng.

[bib0028] Mullangi R., Ahlawat P., Srinivas N.R. (2010). Irinotecan and its active metabolite, SN-38: review of bioanalytical methods and recent update from clinical pharmacology perspectives. Biomed. Chromatogr..

[bib0020] Owusu B.Y., Galemmo R., Janetka J., Klampfer L. (2017). Hepatocyte growth factor, a key tumor-promoting factor in the tumor microenvironment. Cancers. (Basel).

[bib0033] Pek N.M.Q., Liu K.J., Nichane M., Ang L.T. (2021). Controversies surrounding the origin of hepatocytes in adult livers and the in vitro generation or propagation of hepatocytes. CMGH.

[bib0001] Quan Y., Wang Z.Y., Chu X.Y., Zhang H.Y. (2018). Evolutionary and genetic features of drug targets. Med. Res. Rev..

[bib0004] Ram R.N., Gadaleta D., Allen T.E.H. (2022). The role of ‘big data’ and ‘in silico’ New Approach Methodologies (NAMs) in ending animal use – a commentary on progress. Comput. Toxicol..

[bib0010] Ronaldson-Bouchard K., Vunjak-Novakovic G. (2018). Organs-on-a-chip: a fast track for engineered human tissues in drug development. Cell Stem Cell.

[bib0029] Sanghani S.P., Quinney S.K., Fredenburg T.B., Davis W.I., Murry D.J., Bosron W.F. (2004). Hydrolysis of irinotecan and its oxidative metabolites, 7-ethyl-10-[4-N-(5-aminopentanoic acid)-1-piperidino] carbonyloxycamptothecin and 7-ethyl-10-[4-(1-piperidino)-1-amino]-carbonyloxycamptothecin, by human carboxylesterases CES1A1, CES2, and a newly expressed carboxylesterase isoenzyme, CES3. Drug Metab. Dispos..

[bib0002] Scott C.W., Peters M.F., Dragan Y.P. (2013). Human induced pluripotent stem cells and their use in drug discovery for toxicity testing. Toxicol. Lett..

[bib0016] Shinha K., Nihei W., Ono T., Nakazato R., Kimura H. (2020). A pharmacokinetic-pharmacodynamic model based on multi-organ-on-a-chip for drug-drug interaction studies. Biomicrofluidics.

[bib0017] Shinha K., Nihei W., Nakamura H., Goto T., Kawanishi T., Ishida N., Yamazaki N., Imakura Y., Mima S., Inamura K., Arakawa H., Nishikawa M., Kato Y., Sakai Y., Kimura H. (2021). A kinetic pump integrated microfluidic plate (Kim-plate) with high usability for cell culture-based multiorgan microphysiological systems. Micromachines. (Basel).

[bib0032] Shinha K., Nakamura H., Nishikawa M., Sakai Y., Kimura H. (2025). Liver microphysiological system for drug-induced liver injury (DILI) evaluation based on kinetic-pump integrated microfluidic plate (KIM-Plate). IEEJ Trans. Sens. Micromachines.

[bib0022] Stewart A.J., Westley B.R., May F.E.B. (1992). Modulation of the proliferative response of breast cancer cells to growth factors by oestrogen. Br. J. Cancer.

[bib0012] Sung J.H., Kam C., Shuler M.L. (2010). A microfluidic device for a pharmacokinetic – pharmacodynamic (PK – PD) model on a chip †. Lab. Chip..

[bib0024] Tyan S.W., Kuo W.H., Huang C.K., Pan C.C., Shew J.Y., Chang K.J., Lee E.Y.H.P., Lee W.H. (2011). Breast cancer cells induce cancer-associated fibroblasts to secrete hepatocyte growth factor to enhance breast tumorigenesis. PLoS. One.

[bib0027] Uchida K., Otake K., Tanaka K., Hashimoto K., Saigusa S., Matsushita K., Koike Y., Inoue M., Ueeda M., Okugawa Y., Inoue Y., Mohri Y., Kusunoki M. (2013). Clinical implications of CES2 RNA expression in neuroblastoma. J. Pediatr. Surg..

[bib0009] Van Berlo D., van de Steeg E., Amirabadi H.E., Masereeuw R. (2021). The potential of multi-organ-on-chip models for assessment of drug disposition as alternative to animal testing *Curr*. Opin. Toxicol..

[bib0025] Wang C., Gao C., Meng K., Qiao H., Wang Y. (2015). Human adipocytes stimulate invasion of breast cancer MCF-7 cells by secreting IGFBP-2. PLoS. One.

[bib0007] Wang Y., Gao Y., Pan Y., Zhou D., Liu Y., Yin Y., Yang J., Wang Y., Song Y. (2023). Emerging trends in organ-on-a-chip systems for drug screening. Acta Pharm. Sin. B.

[bib0034] Yamasaki C., Ishida Y., Yanagi A., Yoshizane Y., Kojima Y., Ogawa Y., Kageyama Y., Iwasaki Y., Ishida S., Chayama K., Tateno C. (2020). Culture density contributes to hepatic functions of fresh human hepatocytes isolated from chimeric mice with humanized livers: Novel, long-term, functional two-dimensional in vitro tool for developing new drugs. PLoS. One.

[bib0031] Yap J.H., Ishizaki S., Nakamura H., Shinha K., Kimura H. (2025). The fluidic shear stress loading method enables mechanobiological stimulation in an on-chip pump-integrated microphysiological system. Micromachines. (Basel).

[bib0030] Younis I.R., Malone S., Friedman H.S., Schaaf L.J., Petros W.P. (2009). Enterohepatic recirculation model of irinotecan (CPT-11) and metabolite pharmacokinetics in patients with glioma. Cancer Chemother. Pharmacol..

[bib0021] Zhang Y., Moerkens M., Ramaiahgari S., de Bont H., Price L., Meerman J., van de Water B. (2011). Elevated insulin-like growth factor 1 receptor signaling induces antiestrogen resistance through the MAPK/ERK and PI3K/Akt signaling routes. Breast Cancer Res..

[bib0019] Ziegler K.M., Considine R.V., True E., Swartz-Basile D.A., Pitt H.A., Zyromski N.J. (2016). Adipocytes enhance murine pancreatic cancer growth via a hepatocyte growth factor (HGF)-mediated mechanism. Int. J. Surg..

