# Supplementary material for: Metabolism-dependent drug response assessment using a liver-cancer microphysiological system on the kinetic-pump integrated microfluidic plate (KIM-Plate)
Source: NAM J. 2026 Sep 5;2:100127. doi: 10.1016/j.namjnl.2026.100127 (PMC13587913; doi:10.1016/j.namjnl.2026.100127)
Supplement: Supplementary file 1 [file mmc1.docx]

**Supplementary information**

**Table S1.** Experimental design and major differences among the conditions used to evaluate CPT-11 efficacy in the presence of hepatic metabolism. The table summarizes the experimental features of the Well_CM, MPS_CM, and MPS_Coculture conditions and the primary comparisons among these conditions. *Drug and metabolite concentrations during the MCF7 efficacy experiments were not directly measured. The exposure profiles were estimated from CPT-11 metabolic profiles obtained in PXB monoculture experiments. In the CM conditions, the drug and metabolite concentrations present at the beginning of MCF7 exposure were assumed to remain constant during the 72 h exposure period.

| **Experimental feature** | **Well_CM** | **MPS_CM** | **MPS_Coculture** |
| --- | --- | --- | --- |
| Culture platform | 12-well plate | KIM-Plate | KIM-Plate |
| Medium condition | Static | Perfusion | Perfusion |
| PXB cells during MCF7 drug exposure | Absent | Absent | Present |
| PXB-conditioned medium | Present | Present | Not used |
| Hepatic metabolism during MCF7 drug exposure | Absent | Absent | Continuous |
| Drug/metabolite exposure profile | Assumed constant* | Assumed constant* | Time-dependent (estimated)* |
| Continuous PXB–MCF7 exchange | Absent | Absent | Present |
| Primary comparison | Well_CM vs MPS_CM: perfused MPS environment | Reference for both comparisons | MPS_CM vs MPS_Coculture: simultaneous coculture |

**Figure S1.** Schedule for the conditioned medium method on a 12-well plate (Well_CM) and coculture using the KIM-Plate (MPS_Coculture). PXB cells and MCF7 cells were precultured on the 24-well plate. In the MPS_Coculture, cells were introduced into chambers A and B of the KIM-Plate on day 0. In the Well_CM condition, PXB cells were cultured in a 12-well plate starting on day 0. The MCF7 cells were transferred to a 12-well plate on day 3, which coincided with the first medium change for the PXB cells. The culture medium collected from PXB cells on days 3, 5, and 7 was used to change the medium for MCF7 cells on days 3, 6, and 8. KIM-Plate, Kinetic-pump integrated microfluidic plate; MPS, microphysiological systems; CM, conditioned medium.

**Figure S2.** Schedule for drug efficacy tests of CPT-11 considering hepatic metabolism. In the MPS_Coculture condition, cells were introduced on day 0 into chambers A and B of the KIM-Plate. In the CM method, the PXB cells were transferred to a 12-well plate or the KIM-Plate on day 0. The MCF7 cells were transferred to a 12-well plate or the KIM-Plate on day 3. Each cell was cultured in medium containing the drug for the first 3 days, then in drug-free medium for the remaining 4 days. KIM-Plate, Kinetic-pump integrated microfluidic plate; MPS, microphysiological systems; CM, conditioned medium.

**Figure S3.** CPT-11 concentration-cell viability curves in the Well_CM or MPS_Coculture conditions. Cell viability of MCF7 cells in the Well_CM and MPS_Coculture conditions was comparable, regardless of CPT-11 concentration. Data are presented as the mean ± standard deviation (SD). Sample sizes at 1000, 100, and 10 nM CPT-11 were n = 17, 13, and 3 for Well_CM and n = 19, 15, and 4 for MPS_Coculture, respectively. MPS, microphysiological system; CM, conditioned medium.

**Figure S4.** Area under the curve (AUC)-cell viability curves in monoculture of MCF7 cells. Cell viability was higher under concentration-change conditions than under constant-concentration conditions, despite similar AUCs. Data are presented as the mean ± standard deviation (SD), with n = 4–6 for all conditions.

**Supplemental Experiment 1**

MCF7 cells were exposed to SN-38 for 3 days in a 48-well plate and cultured in SN-38-free medium for 4 days. The culture medium was changed every 24 h throughout the drug exposure period. In the constant-concentration conditions, the cells were exposed to the same concentration for 3 days. Under concentration change conditions, cells were exposed to higher concentrations of SN-38 when the medium was changed. For example, to achieve the same area under the concentration-time curve (AUC) of SN-38 after exposure to 14 nM SN-38 for 3 days, the drug concentrations were modified as follows: day 1; 6 nM, day 2; 6 nM, day 3; 30 nM or day 1; 3 nM, day 2; 9 nM, day 3; 30 nM.

Under concentration-change conditions, cell viability gradually approached 50% as the AUC increased (Figure S4). In contrast, under constant-concentration conditions, cell viability reached 50% at a lower AUC than under concentration-change conditions. GI_50_ values calculated from these results were 192.5 nM·h under constant-concentration conditions and 633.4 nM·h under concentration-change conditions.

**Table S2.** Number of experimental replicates used for statistical analyses. The reported n values were obtained from independently cultured wells or KIM-Plate units. Except for the drug-concentration measurements, experiments were performed on multiple separate experimental occasions. Drug-concentration measurements were obtained from independently cultured wells or KIM-Plate units within a single culture experiment.

| **Figure** | **Condition** | **Drug / Concentration** | **n** | **Statistical analysis** |
| --- | --- | --- | --- | --- |
| 1 (D and E) | Well_Monoculture | --- | 11 | Welch’s t-test |
|  | Well_CM | --- | 10 |  |
|  | MPS_Monoculture | --- | 11 |  |
|  | MPS_Coculture | --- | 12 |  |
| 2 (A-C) | Well | CPT-11, SN-38, SN-38G | 3 | Welch’s t-test |
|  | MPS | CPT-11, SN-38, SN-38G | 3 |  |
| 3 (A, C and G) | Well | CPT-11 / 0, 100, 1,000 nM | 14 | Two-way ANOVA followed by Tukey |
|  |  | CPT-11 / 10 nM | 3 |  |
| 3 (B, C and G) | MPS | CPT-11 / 0 nM | 14 | Two-way ANOVA followed by Tukey |
|  |  | CPT-11 / 10 nM | 3 |  |
|  |  | CPT-11 / 100, 1,000 nM | 15 |  |
| 3 (D, F and H) | Well | SN-38 / 0 nM | 7 | Two-way ANOVA followed by Tukey |
|  |  | SN-38 / 1, 100 nM | 5 |  |
|  |  | SN-38 / 10 nM | 4 |  |
|  |  | SN-38 / 1,000 nM | 3 |  |
| 3 (E, F and H) | MPS | SN-38 / 0 nM | 7 | Two-way ANOVA followed by Tukey |
|  |  | SN-38 / 1, 10, 100 nM | 6 |  |
|  |  | SN-38 / 1,000 nM | 3 |  |
| 4 (A-C) | Well_CM | CPT-11, SN-38 | 3 | One-way ANOVA followed by Tukey |
|  | MPS_CM | CPT-11, SN-38 | 3 |  |
|  | MPS_Coculture | CPT-11, SN-38 | 3 |  |
| 4 (E) | Well_CM | CPT-11 | 17 | Two-way ANOVA followed by Tukey |
|  | MPS_CM | CPT-11 | 10 |  |
|  | MPS_Coculture | CPT-11 | 19 |  |
